# Exploratory Movement Generates Higher-Order Information That Is Sufficient for Accurate Perception of Scaled Egocentric Distance

**DOI:** 10.1371/journal.pone.0120025

**Published:** 2015-04-09

**Authors:** Bruno Mantel, Thomas A. Stoffregen, Alain Campbell, Benoît G. Bardy

**Affiliations:** 1 Movement-to-Health Laboratory, EuroMov, Montpellier-1 University, Montpellier, France; 2 Normandie Université, Caen, France; 3 Centre d’Etudes Sport et Actions Motrices, Université de Caen Basse-Normandie, Caen, France; 4 Affordance Perception-Action Laboratory, University of Minnesota, Minneapolis, United States of America; 5 UMR 6139 Laboratoire de Mathématiques Nicolas Oresme, Université de Caen-Basse Normandie & CNRS, Caen, France; 6 Institut Universitaire de France, Paris, France; Centre de Neuroscience Cognitive, FRANCE

## Abstract

Body movement influences the structure of multiple forms of ambient energy, including optics and gravito-inertial force. Some researchers have argued that egocentric distance is derived from inferential integration of visual and non-visual stimulation. We suggest that accurate information about egocentric distance exists in perceptual stimulation as higher-order patterns that extend across optics and inertia. We formalize a pattern that specifies the egocentric distance of a stationary object across higher-order relations between optics and inertia. This higher-order parameter is created by self-generated movement of the perceiver in inertial space relative to the illuminated environment. For this reason, we placed minimal restrictions on the exploratory movements of our participants. We asked whether humans can detect and use the information available in this higher-order pattern. Participants judged whether a virtual object was within reach. We manipulated relations between body movement and the ambient structure of optics and inertia. Judgments were precise and accurate when the higher-order optical-inertial parameter was available. When only optic flow was available, judgments were poor. Our results reveal that participants perceived egocentric distance from the higher-order, optical-inertial consequences of their own exploratory activity. Analysis of participants’ movement trajectories revealed that self-selected movements were complex, and tended to optimize availability of the optical-inertial pattern that specifies egocentric distance. We argue that accurate information about egocentric distance exists in higher-order patterns of ambient energy, that self-generated movement can generate these higher-order patterns, and that these patterns can be detected and used to support perception of egocentric distance that is precise and accurate.

## Introduction

Animate movement alters the structure of multiple forms of ambient energy. Consider walking. As the feet strike the surface of support this inertial contact alters the stimulation of pressure sensors in the skin, of receptors in the joints, of stretch receptors in the muscles, as well as dynamic patterns of gravitoinertial force at the vestibular system. If the environment is illuminated, walking will alter patterns of optic flow. The patterns that are created in optics and inertia are not identical; they are non-redundant. Traditionally, the existence of non-redundancy in patterns of simultaneous multimodal stimulation arising from animate movement have been interpreted within the epistemological assumptions of indirect perception [1–5]. In theories of indirect perception it is assumed that patterns of stimulation available to the perceiver bear an ambiguous relation to physical reality. If this is true, then accurate perception can occur only as a product of inferential processing within the nervous system. In the case of multimodal stimulation the required processing is assumed to entail some type of integration of disparate inputs from different senses. In the present contribution, we offer an interpretation that is consistent with the epistemological assumptions of direct perception. In theories of direct perception it is argued that patterns of stimulation available to the perceiver bear a unique, lawful relation to physical reality [6–10]. If this is true, and if perceivers are sensitive to the relevant patterns of stimulation, then sensory stimulation may be sufficient for accurate perception, such that inferential processing is not required. We argue that the epistemological assumptions of direct perception can apply to the multisensory consequences of animate movement [6].

In this article, we focus on the perception of scaled egocentric distance (sometimes referred to as absolute distance, e.g., [11]). Perception of egocentric distance tends to be very poor for stationary perceivers, but often is greatly improved when perceivers are allowed to move [12,13]. How might this superior performance be achieved? Traditional analyses of multisensory stimulation have focused exclusively on patterns that exist within individual forms of ambient energy, such as patterns of optic flow, patterns of acoustic stimulation, and so on. In such analyses relations between different senses can exist only as products of internal processing. Taking a qualitatively different approach, we consider the possibility that accurate information may exist in patterns that extend across multiple forms of ambient energy. We quantify analytically an emergent, higher order parameter that extends across optic and inertial energies, and which is related to egocentric distance. We report an experiment in which we manipulated the availability of this parameter independent of parameters that were available to individual perceptual systems. Our results are consistent with the hypothesis that participants detected and used the higher order parameter, rather than internal processing to inputs derived from individual perceptual systems.

An important methodological feature of our study concerns the types of exploratory movement that are available to perceivers. In previous research on the perception of egocentric distance experimental participants typically have been limited to self-generated movement in one or two dimensions. In the experiment that we report participants were free to move in any direction(s) that they wished. This methodological factor is important because the higher order parameter that we identify is generated by movement of the perceiver in three dimensions. For this reason, our study is the first empirical research in which it has been possible to evaluate the perceptual reality of the higher order parameter.

### Analysis of available information

Displacement of the head relative to the illuminated environment generates optic flow. Displacement of the head relative to the gravito-inertial force environment generates changes in ambient forces. Thus, self-generated head movements yield simultaneous stimulation of the visual, vestibular, and kinesthetic systems. Relations between patterns in optics and in gravito-inertial forces depend upon relations between head movements relative to the illuminated and gravito-inertial environments.

Early models of the information about egocentric distance available in optic flow have been restricted to one-dimensional movements [11,14–16] and sometimes further limited in terms of direction, frequency, or amplitude of movement [17,18]. For example, when the point of observation moves along a rectilinear trajectory, the distance to a stationary environmental object can be expressed as a function of the (optical) angle between the axis of motion and the direction of the object, the (optical) speed at which this angle changes and the (non optical) velocity of the point of observation [14] (see also [11,16,17]):

$$D=\frac{v\cdot \sin\alpha }{\dot{\alpha }}$$ (1)

with $v=\left\|\vec{v}\right\|$. Below, we extend these analyses to include natural, unrestricted head movements. As a preliminary step, we first consider the case of any 2D movements performed within the plane of the object.

### Information about egocentric distance in the case of 2D head movements

**Fig 1:**
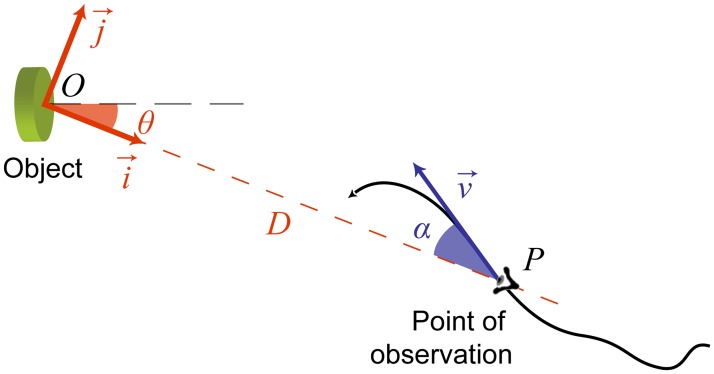
Optical and non-optical consequences of a movement executed within the plane of a stationary object. The egocentric distance can be expressed as a function of directional parameters (*α*, *θ*) and linear parameters about head movements (*v*).

Let $\left(\vec{i},\vec{j}\right)$ be a mobile orthonormal basis lying within the plane of movement. The basis is always centered on the stationary object *O* but its orientation changes such that $\vec{i}$ is always pointing toward the point of observation *P*, and makes an angle *θ* with the axis of another basis whose direction is fixed relative to the object/earth (Fig 1). The velocity $\vec{v}$ of the point of observation makes an angle *α* with the direction of the object and thus can be decomposed as

$$\vec{v}=v\cdot \;\left(\cos\alpha \;\vec{i}+\sin\alpha \;\vec{j}\right)$$ (2)

The velocity can also be expressed as a function of the distance *D* between the point of observation and the object,

$$\vec{v}=\frac{d\;\vec{OP}}{dt}=\frac{d\;D\;\vec{i}}{dt}=\dot{D}\;\vec{i}+D\;\dot{\theta }\;\vec{j}$$ (3)

By combining Eqs 2 and 3 and projecting on unit axis $\vec{j}$ we obtain

$$D=\frac{v\cdot \;\sin\alpha }{\dot{\theta }}$$ (4)

where *D* is the distance between the point of observation and the object, *v* is the norm of the linear velocity $\vec{v}$ of the point of observation *P*, *α* is the angle between $\vec{v}$ and the direction of the object relative to *P*, and $\dot{\theta }$ is the angular velocity at which the direction of *P* changes relative to the object, or equivalently (by symmetry) the angular velocity at which the direction of the object changes relative to *P* (i.e., so-called apparent motion of the target).

The above analysis indicates that scaled egocentric distance is also specified to a perceiver moving in 2D in his/her optic-inertial stimulation. As opposed to $\dot{\alpha }$ in the 1D model (Eq 1; [11,14–16]), in the 2D model, the optical velocity parameter $\dot{\theta }$ is no longer defined relative to the direction toward which the point of observation is moving.

### Higher order specification of egocentric distance in the case of 3D head movements

To extend Eq 4 to the case of unrestrained three-dimensional movements, a first intuitive approach is to consider that the plane which contains $\vec{v}$ and $\left(\vec{i},\vec{j}\right)$ is itself rotating relative to the earth (about *O*) and, accordingly, to introduce two more angles (*φ* and *ψ*) to characterize the orientation of that plane relative to an earth-fixed reference frame (see S1 Fig and S1 Text for details):

$$D=\frac{v\cdot \sin\alpha }{\dot{\theta }+\dot{\psi }\cos\varphi }$$ (5)

Although intuitive for describing three-dimensional movements, the decomposition of optical motion in three rotations (*θ*, *φ* and *ψ*) about arbitrary axes is not necessarily relevant for a perceiver interacting with his/her environment. In addition, the description obtained within that framework also indicates that the three angular parameters are related (see S1 Text):

$$\;-\dot{\psi }\cos\theta \;\sin\phi +\dot{\phi }\sin\theta =0,$$ (6)

suggesting that a more circumspect description can be reached. A first solution, which allows us to use two rotations instead of three, is to use a spherical coordinate system in place of the Cartesian one (see S1B Fig and S1 Text for details):

$$\left|D\right|=\frac{v\cdot \left|\;sin\;\left(\alpha \right)\;\right|}{\sqrt{\;{\dot{\varphi }}^{2}cos^{2}\delta +{\dot{\delta }}^{2}}}$$ (7)

However, as above, decomposing angular motion about two arbitrary axis is not satisfactory. An alternative solution, which allows for a more parsimonious description can be obtained from the definition of the cross-product:

$$\left\|\;\vec{i}\wedge \vec{v}\;\right\|=v\cdot \left|\;\sin\;\left(\alpha \right)\;\right|$$ (8)

From Eq 3 we also have,

$$\left\|\;\vec{i}\wedge \vec{v}\;\right\|=\left\|\;\vec{i}\wedge \left(\dot{D}\;\vec{i}+D\;\frac{d\;\vec{i}}{dt}\right)\;\right\|=\left\|\;\vec{i}\wedge D\;\frac{d\;\vec{i}}{dt}\;\right\|$$ (9)

By combining Eqs 8 and 9 we can thus express the distance between a perceiver performing any 3D movement and a stationary object:

$$\left|D\right|=\frac{v\cdot \left|\;\sin\;\left(\alpha \right)\;\right|}{Q}$$ (10)

where *v* is the norm of the velocity of the point of observation *P*, *α* is the angle between the direction of movement and the direction of the object relative to *P*, and *Q* is the norm of a rotational vector $\vec{\Omega }$, characterizing the change in the direction of *P* relative to the object (or, symmetrically, of the object relative to *P*):

$$Q=\left\|\vec{\Omega }\right\|=\left\|\;\vec{i}\cdot \frac{d\vec{i}}{dt}\;\right\|$$

with $\vec{i}$ the unit vector of a mobile base, pointing from the object toward the point of observation (see Fig 1). As $\dot{\theta }$ in the 2D model (Eq 4), but as opposed to $\dot{\alpha }$ in the 1D model (Eq 1; [11,14–16]), *Q* is not defined relative to the direction toward which the point of observation *P* is moving; rather it is the rate of change of the direction of *P* relative to the object (or symmetrically of the object relative to *P*). According to Eq 10 the distance *D* is a relational property of optical (*α*, *Q*) and non optical ($\vec{v}$) dimensions of the stimulation (*Q* can also be viewed as the speed at which the eye/head must counter rotate in order to maintain the object at the same location within the field of view). Eqs 1, 4, and 10 can also be differentiated to express distance as a function of higher order derivatives (acceleration, jerk, etc.). For example, the two successive time derivatives of Eq 10 yield

$$D=\frac{\dot{v}\sin\alpha +v\cos\alpha \left(\dot{\alpha }-Q\right)}{\dot{Q}}$$ (11)

and

$$D=\frac{\ddot{v}\sin\alpha +\left(\dot{v}\;\left(2\dot{\alpha }-Q\right)+v\;\left(\ddot{\alpha }-2\dot{Q}\right)\right)\cos\alpha -v\dot{\alpha }\left(\dot{\alpha }+Q\right)\sin\alpha }{\ddot{Q}}$$ (12)

Interestingly, Eqs 10–12 assume simpler forms for particular trajectory shapes. For example, if the perceiver’s movement is rectilinear or plane, then Eq 10 simplifies into Eq 1 and Eq 4, respectively. The formal description also simplifies when the perceiver’s movement is orthogonal to the direction of the target (i.e., when movement is on a sphere centered on the target). Within optic flow, this type of movement generates translatory motion and perspective changes but no looming or receding motion. In that case, *α* = 90°, sin(*α*) = 1 and Eq 10 simplifies into:

$$D=\frac{v}{Q}$$ (13)

and its two first time derivatives are

$$D=\frac{\dot{v}}{\dot{Q}}$$ (14)

$$D=\frac{\ddot{v}}{\ddot{Q}}$$ (15)

Importantly, these simplifications do not necessitate that the whole trajectory be rectilinear or orthogonal to the target, but only that it be at least locally (and approximately) rectilinear, plane or orthogonal (e.g., tangential). For instance, Eq 13 provides a consistent approximation of the actual distance of the object even when the direction of head movements (relative to the target) deviate from pure orthogonality: If the deviation is equal to ±15°, the distance specified will overestimate the actual distance by only 3.4% (i.e., 3.4 cm if the object is at 1 m, 6.8 cm if it is 2 m far, etc.). Whereas 2-d movement yielded ambiguity, our analysis shows that 3-d movement yields specification in an emergent, higher order pattern. Thus, our analysis reveals that, in principle, movement of the observer generates patterns in ambient energy that are sufficient for accurate, non-inferential perception of egocentric distance.

### Exploratory activity and the pickup of information

In Eqs 1, 4–5, 7,10–12,13–15, *v* and its derivatives influence the stimulation of the vestibular and kinesthetic systems, while *α*, *θ*, *Q* and their derivatives influence the stimulation of the visual system. Determinate information about egocentric distance is available only in relations between stimulation available to these perceptual systems. That is, the information is an emergent property that does not exist in the stimulation available to any individual perceptual system. The equations do not impose any particular metrics. The unit in which they specify egocentric distance depends on the unit used to describe head kinematics (e.g., conventional or intrinsic).

If the perceiver is stationary relative to the illuminated environment, the gravito-inertial environment or both, then Eqs 1, 1, 4–5, 7,10–12,13–15 are undefined or ineffective. Therefore, specification of egocentric distance requires not only optic flow, but also movements of the head and/or body relative to the gravito-inertial environment. The differences among equations further underline that the form of the intermodal pattern specifying distance depends on the characteristics of the movement performed by the perceiver. As a consequence, the perceptual skills required to perceive distance could vary as a function of the exploratory activity of the perceiver. For example, when the point of observation is moving at constant speed (i.e., linear acceleration $\dot{v}=0$), some equations simplify (e.g., Eqs 11–12, dedicated to any 3D movements) while some others are ineffective (e.g., Eq 14, dedicated to movements orthogonal to the direction of the target). Hence, if a perceiver is not sensitive to linear acceleration, then moving at constant speed can allow him/her to exploit Eqs 11–12 to perceive distance. On the other hand, if the perceiver is sensitive to linear acceleration, then moving at constant speed would not change his/her ability to exploit the Eqs 11–12, but would prevent him/her from exploiting Eq 14. In a similar way, if the perceiver’s movement is roughly orthogonal to the direction of the target, then the description of available information about distance simplify into Eqs 13–15. In these equations, egocentric distance is no longer a function of parameter *α* and its derivatives. In other words, with such movements, distance perception no more depends on the perceiver’s sensitivity to the direction of the target relative to his/her direction of movement.

Outside the laboratory, situations in which an actor is completely static or moving at constant velocity are rare. However, we can predict a failure to perceive scaled egocentric distance if the actor’s movement and the resulting apparent optical motion of the object were too low (or with a too low acceleration, jerk, etc.) relative to the object’s distance. More generally, the formal analysis suggests that using particular movements could ease (or impede) information pick up.

Existing experimental work that has addressed the perception of egocentric distance systematically has restricted participants to rectilinear movements. The majority of these studies have evaluated the role of head translations in two directions: either toward the target, e.g., [13,19–22], or in the orthogonal direction—laterally—[12,18,22–24]. Other studies have examined slightly diagonal translation [25] or rotation of the head about its longitudinal axis [26]. In many cases, experimenters have instructed participants to perform “regular, repetitive”, “oscillatory” or “rhythmic” movements, e.g., [19,22,26], and have restricted exploratory movements to specific amplitudes and frequencies, e.g., [12,13,18,23]. These restrictions on movement may have been convenient analytically, but are difficult to justify in terms of natural behavior outside the laboratory (except when the point of observation moves together with an aircraft, as in [14]). Moreover, as we underlined, artificial restrictions on exploratory behavior necessarily constrain the nature and availability of emergent intermodal information about egocentric distance. It is worth noting that Eq 1, which pertains to rectilinear movements, applies identically to rectilinear movements in any direction, whether along the line of sight, perpendicular to it, or any intermediate direction.

In the experiment reported below, we allowed participants to move freely, provided only that they remained seated. Permitting free movement made it possible for us (i) to evaluate human’s ability to perceive egocentric distance when the form under which information is available is not artificially restricted by the experimenter and (ii) to analyze participants’ self-selected exploratory activity.

### Experiment

**Fig 2:**
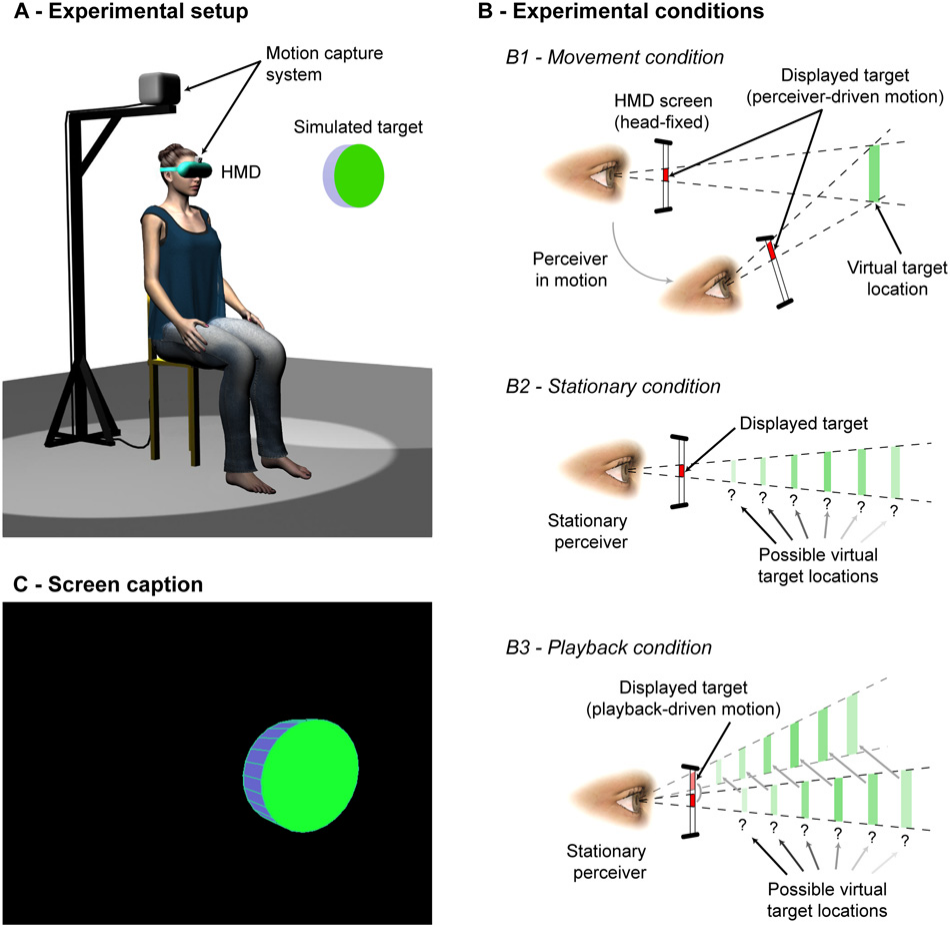
Experimental design. (A) Set up. The participant’s head position and orientation were sampled and used in real time to drive the display of a HMD so as to depict a stationary virtual target. (B) Experimental conditions (see text for details). (C) Screen capture. The virtual target was the only visible element on the HMD screen.

We formalized above an intermodal invariant, *Ii*, specifying the egocentric distance of a static object for a moving perceiver. That information is available in the structure of ambient energy does not imply that it is actually detected. To evaluate whether *Ii* can be picked up and used by humans, we conducted the following experiment. Seated participants were asked to judge (yes/no) whether a visible object was within reach. We manipulated the relation between optics and haptics/inertia that was available to participants during the judgment task. We used a virtual environment system in which an optical display could be updated in real time on the basis of data about displacement of the head in the gravito-inertial space. In addition to manipulating *Ii*, the virtual set up allowed to control all other potential information about egocentric distance. Fig 2 illustrates the experimental set up (A), the experimental conditions (B) and shows a screen capture illustrating the participants view during the experiment (C). We hypothesized that egocentric distance would be perceived accurately when the natural congruence between sources was preserved, that is, when Equation Eqs 1, 1, 4–5, 7,10–12,13–15 were defined. Hereafter, this intermodal condition is called the Movement condition (Fig 2B1) because participants were allowed to freely explore the scene by moving their head relative to the virtual object prior to giving their judgment. We also hypothesized that performance in the Movement condition would contrast with the performance in two control conditions in which optic flow was presented alone (i.e., in the absence of coordinated inertial stimulation). In the Stationary condition, participants were stationary while looking at a static display of the object (Fig 2B2). In the Playback condition, participant also remained still, but the display of the object was driven by his/her own previously recorded movements, recorded during earlier (Movement) trials (Fig 2B3).

Participants were presented targets at 17 different distances. To control for potential range effects, e.g., [27,28], participants were randomly split into two groups. For the Near group, target distances ranged from 23 to 135% of the actual maximum reachable distance (MR_A_, measured prior to the experiment for each participant, see Material and Methods section). For the Far group, distances ranged from 72 to 184%. Our primary dependent variables were the accuracy and precision of the judgments about whether stimulus objects were within reach. These were derived from the psychometric curves fitted to the data and were respectively indicators of the constant and variable error in judgments. We also analyzed exploratory head movements in different conditions as an indicator of whether and how participants exploited Eqs 1, 4, 10–12, 13–15.

## Results

### Reachability judgments

**Fig 3:**
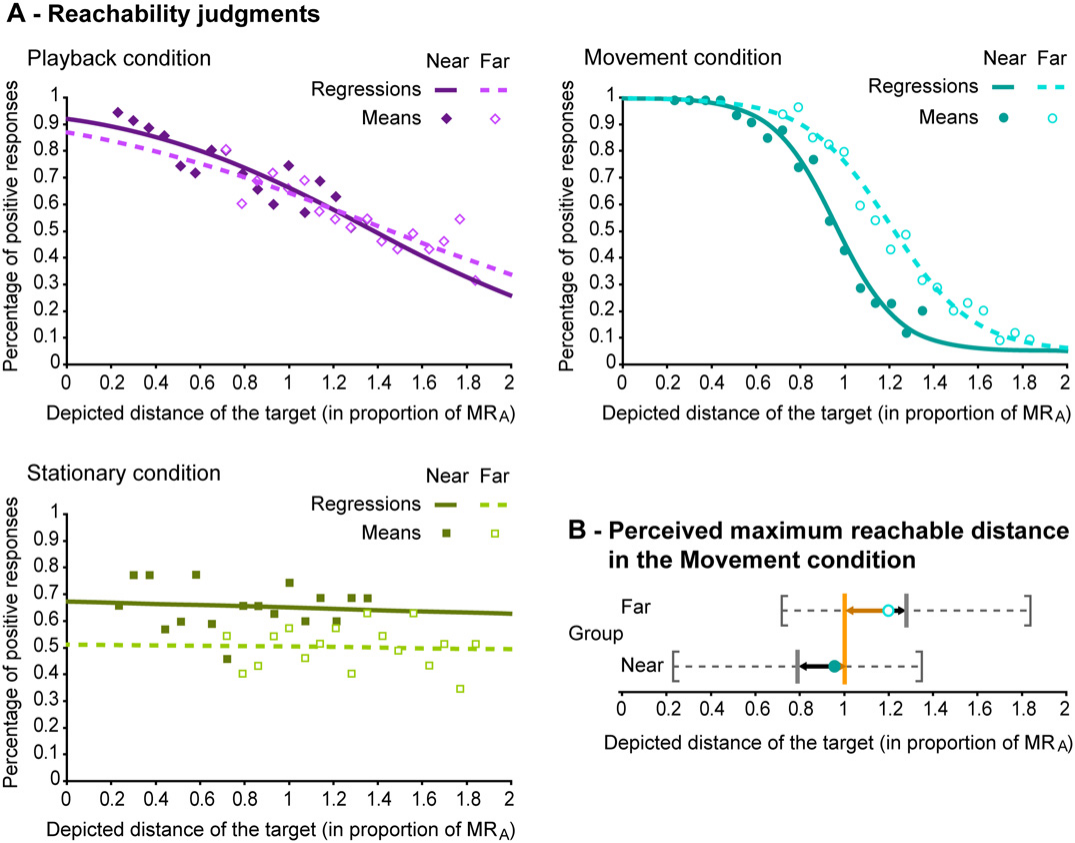
Reachability judgments. (A) Global regression curves (lines) and means (symbols) for both groups of participants in Movement (blue), Stationary (violet) and Playback conditions (green). The percentage of positive responses is plotted as a function of target distance (expressed in proportion of the actual maximum reachable distance, MR_A_). (B) Perceived maximum reachable distance MR_P_ for each group (Near and Far, respectively dark and light blue dots) in the Movement condition. The vertical grey bars mark the median of the set of tested distances for each group of participant (the range of tested distances is indicated by the grey brackets and dotted lines). The vertical orange bar represents MR_A_ for both groups. In each group, MR_P_ is biased toward the median (black arrows) but simultaneously attracted toward MR_A_, as expected (brown arrows).

The deviance test, assessing goodness-of-fit, showed that all the global regressions were good summaries of the corresponding group data, thus ensuring that the derived parameters were relevant. The global regression curves for all experimental conditions (Movement, Stationary and Playback) are plotted in Fig 3A, showing the evolution of the proportion of “yes” judgments as a function of increasing simulated distance. Individual regressions were used to compare performance across conditions. Individual fits for which the deviance test was not significant were not included in the analyses. In the Stationary condition, the frequent absence of consistency in judgments yielded only 7 reliable individual fits. Means and standard deviations of slopes and perceived maximum reachable distance (MR_P_) for significant individual fits are given in S1 Table. Because of the non-normal distribution of slopes and absolute errors from individual regression across conditions, we compared them with Wilcoxon non-parametric tests.

In the Movement condition, the slopes obtained from the global regression were -1.76 and -1.33, respectively for the Near and Far groups (*CI*_.*95*_ = -2.15, -1.45 and *CI*_.*95*_ = -1.68, -1.09). This meant that when the target’s distance was increased from -20% to + 20% of MR_A_ (i.e., approximately -12cm to +12cm), it caused reachability judgments in the Movement condition to drop from 78% to 26% of “yes” (i.e., reachable). By contrast, these slopes were 4 times smaller (-0.45 and -0.34) in the Playback condition (*CI*_.*95*_ = -0.60, -0.26 and *CI*_.*95*_ = -0.47, -0.19, for Group Near and Far), and more than 60 times smaller (-0.03 and -0.01) in the Stationary condition (*CI*_.*95*_ = -0.17, 0.00 and *CI*_.*95*_ = -0.15, 0.00). Wilcoxon tests confirmed that the precision of judgments was significantly lower for Movement than for the two other conditions: between Movement and Stationary (*Z*(*N* = 7) = 2.20, p <. 05, *d*_*w*_ = -0.71), between Movement and Playback (*Z*(*N* = 13) = 3.18, p <. 005, *d*_*w*_ = -1.00). Conversely the difference between Stationary and Playback was not significant (*Z*(*N* = 7) = 1.18, ns, *d*_*w*_ = -0.71). Altogether, these results indicate that reachability judgments in the Movement condition were far more precise than those in the Playback and Stationary conditions.

In the Movement condition, participants from the Near group underestimated their actual reaching capabilities, while participants in the Far group overestimated their actual reaching abilities. Each shift was in direction of the middle of the sets (Fig 3B, black arrows), suggesting a classic centering bias, that is, a bias toward the middle of the testing interval, e.g., [28]. Despite the absence of prior training or knowledge, participants appear to have calibrated their judgments relative to the sets of distances, as if they were roughly balancing the number of their positive and negative judgments. Nevertheless, the perceived maximum reachable distance MR_P_ was attracted by the actual maximum reachable distance MR_A_ (Fig 3B, brown arrows). When averaged over participants, the absolute error of judgments in the Movement condition was 20.3% of MR_A_ (*CI*_.*95*_ = 11.4, 29.1), which corresponded to 12.5cm. By contrast, the mean absolute errors in the Stationary and Playback conditions, respectively 114.6% and 111.1%, were more than 5 times larger (*CI*_.*95*_ = 51.7, 177.6 and *CI*_.*95*_ = 0.0, 234.5). This difference between Movement and the two others condition was confirmed by the Wilcoxon tests conducted on individual absolute error values, *Z*(*N* = 7) = 2.37, *p* <. 05, *d*_*w*_ = 1.00 and *Z*(*N* = 13) = 2.27, *p* <. 05, *d*_*w*_ = 0.38, respectively for comparison with Stationary and Playback. The difference between Stationary and Playback was not significant (*Z*(*N* = 7) = 0.68, *ns*, *d*_*w*_ = -0.43). As predicted, judgments made when the *Ii* was available (Movement condition) were more accurate than when it was not (Stationary and Playback conditions).

### Confidence ratings

In addition to judging whether the target was reachable, participants also rated between 1 (low) and 5 (high) how confident they were about their judgments. Following previous studies, e.g., [29], we hypothesized that confidence ratings would offer a converging indicator of the participants’ ability to perceive whether the targets were within reach or not. We expected that, when distance information was available (i.e., in the Movement condition), participants would be more confident about their judgments when the target was very near or very far (unambiguous situation) than when the target was close to the reachable/not reachable boundary (ambiguous situation). Conversely, we anticipated that no such trend would emerge in the two other conditions. This U-shape hypothesis can also be understood from a dynamical systems perspective [30]: in that case, the actual maximum reachable distance corresponds to a transition point between two stable regimes, which represent two different action modes (e.g., reaching with the arm only vs. reaching with the arm plus leaning the torso forward). From that point of view, the lower confidence of participants would result from the larger susceptibility to noise and from the increase of the variability of the order parameter, exhibited by such nonlinear systems at regime boundaries.

**Fig 4:**
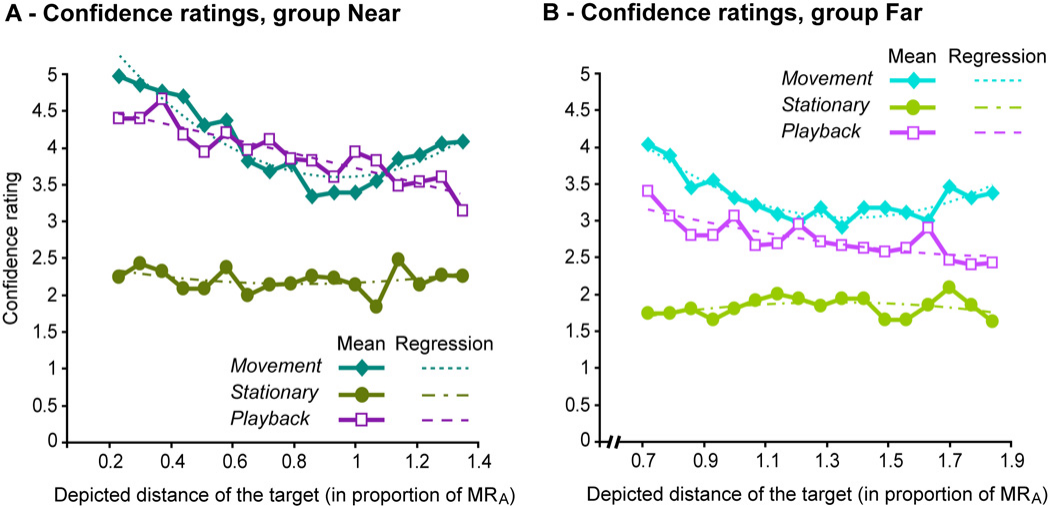
Mean confidence ratings expressed as a function of target distance. The rating scale ranged from 1 (lowest) to 5 (highest). (A) Near Group. (B) Far Group. Distance on abscissa axis is expressed in proportion of the actual maximum reachable distance, MR_A_. Dotted lines represent second order polynomial regressions (see text and S2 Table for details).

The data are summarized in Fig 4. As expected, when plotted as a function of target distance, ratings exhibited U-shaped curves in the Movement condition (with an additional slight asymmetry between near and far judgments), whereas the curves were flat in the Stationary condition and exhibited a constant decrease in the Playback condition. To quantify these trends we fitted 2^nd^ order polynomials to the rating curves for each group and in each condition (full equations, *R^2^* and parameters statistics are given in S2 Table). The parameters associated to the *x^2^* terms (quantifying the ‘openness of the U’) were of 3.18 and 2.15 for the Near and Far groups in the Movement condition, while they did not exceeded 0.51 in the two other conditions. Among the six regressions, these parameters were only significant for the Movement condition.

### Exploratory movements

We also analyzed the exploratory movements that participants used to create information about distance in the Movement condition. The mean amplitude of head displacement along the principal axis of movement was 1.7 cm (*CI*_.*95*_ = 1.6, 1.9) in the Playback condition, and 1.4 cm (*CI*_.*95*_ = 1.3, 1.5) in the Stationary condition, as compared with 26.5 cm (*CI*_.*95*_ = 25.9, 27.1) in the Movement condition. For this reason, and because in the two conditions without movement the display was not driven by participants’ movement, we limited our analysis of exploratory movement to the Movement condition.

**Fig 5:**
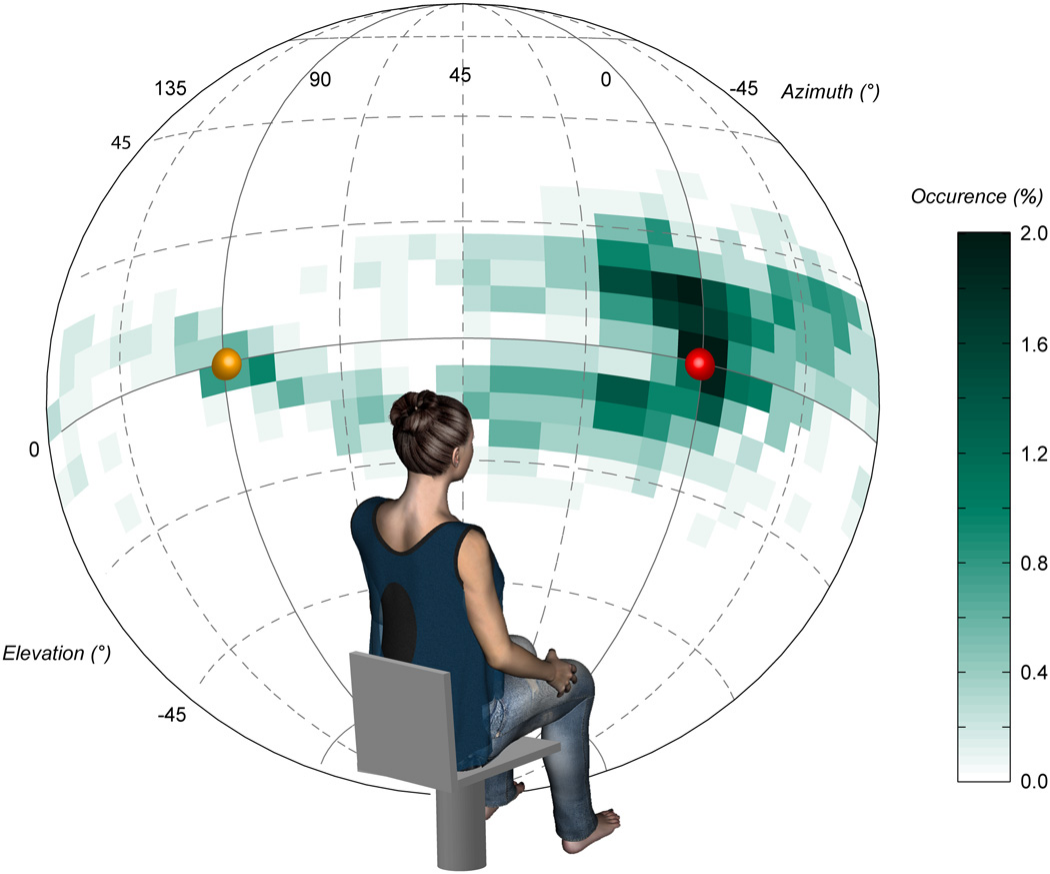
Frequency plot of principal directions of movement for all trials and participants in Movement condition. The principal direction corresponded to the eigenvector associated with the largest eigenvalue of the trial orientation matrix. A principal direction was computed for each trial, but only those for which the eigenvalue exceeded. 5 and the ratio of second and largest eigenvalues was less than. 8 were included in the plot (1147 out of the 1190 trials). The red and orange dots represent the two directions usually tested in the literature: toward the target (AP) and laterally (ML), respectively.

As we have seen, formal descriptions of *Ii* (e.g., 1, 4, 10–12, 13–15) depend on the characteristics of the perceiver’s movement (e.g., 1D, 2D or 3D; orthogonal to the direction of the target). Previous empirical investigations systematically restricted exploratory behavior in terms of dimension (1D) and direction (lateral or toward the target). As our participants were not imposed any specific trajectory, we first wondered whether they would spontaneously use 1D displacements, and if so, whether these movements would be oriented toward either of the two directions usually tested in the literature (lateral or toward the target). To that end, we analyzed the distribution of instantaneous directions of movement during each trial. The eigenvalues of the orientation matrix provide a measure of the variance in movement direction explained by each of the corresponding eigenvectors. When averaged over participants and trials, the three eigenvalues were respectively 68.1% (*CI*_.*95*_ = 67.6, 68.6), 23.6% (*CI*_.*95*_ = 23.1, 24.1) and 8.3% (*CI*_.*95*_ = 8.2, 8.5). That the second and smallest eigenvalues accounted for more than 30% of the total variability indicates that movements were not merely one-dimensional (rectilinear). For each trial, the eigenvector associated with the largest eigenvalue provided a measure of the principal direction about which head trajectory was organized. As illustrated in the frequency plot in Fig 5, these principal directions were more often heading toward the target (red point) than perpendicular to it (orange point). However, they were not merely heading straight toward the target; rather they were spread over a wide range of diagonal directions, which differed among participants and to a smaller extent across trials for a same participant. Hence, as can be seen on Fig 5 from the comparison between the green cloud and the red and orange points, our participants’ self chosen patterns of movement contrasted with the two unique directions usually imposed in the literature, e.g., [12–14,18–23].

Although the equations we formalized specify egocentric distance for any amplitude of movement and any magnitude of head speed (or acceleration, jerk, etc.), the perceiver’s ability to pick up *Ii* could impose specific constraints on exploratory movement. In particular, for a same movement of the perceiver, the resulting speed (and acceleration, jerk, etc.) at which the direction of the object changes relative to the eye decreases as the distance to the (virtual) target increases. A limit case would be that of objects in the horizon, which are so far that the change in their direction relative to the eye induced by head movements is marginal (and imperceptible). To use *Ii*, a perceiver must keep its salience within stimulation and thus has to move in a way that maintains a certain minimal amount of angular speed, acceleration and/or jerk in optic flow. To do so, when the distance to the object increases, the perceiver can increase his/her movement amplitude (when it results in getting closer to the target, increasing movement amplitude yields higher optical speed, acceleration and jerk for a given head movement), speed, acceleration, and/or jerk, depending on whether he/she is using first, second and/or third order-based information.

**Fig 6:**
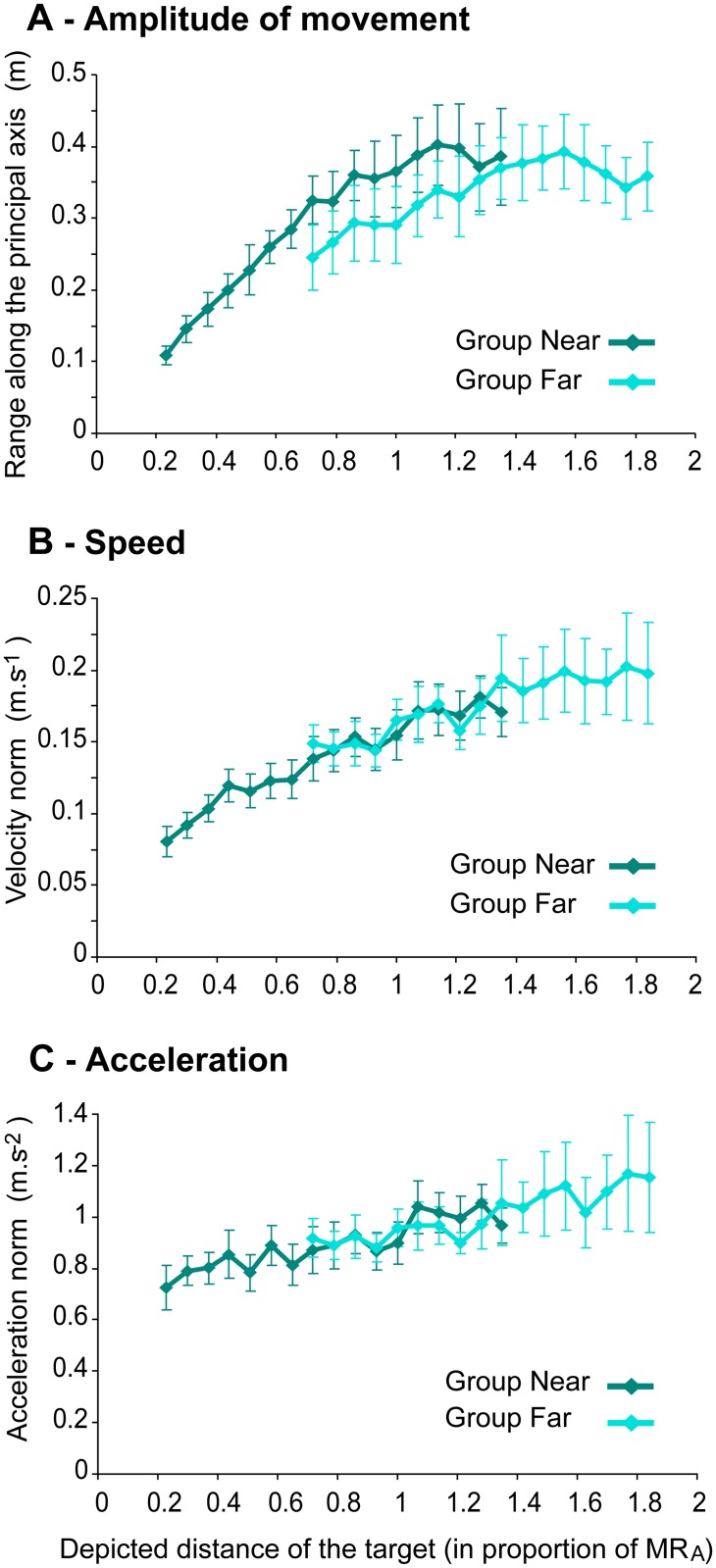
Amplitude, instantaneous speed, and acceleration of head movements in the Movement condition. Mean values with standard errors are plotted as a function of initial target distance, expressed in proportion of the actual maximum reachable distance, MR_A_. (A) Mean range along principal axis. (B) Mean norm of instantaneous velocity. (C) Mean norm of instantaneous acceleration.

Fig 6 shows the evolution of the average movement amplitude (A), as well as the evolution of the average norm of instantaneous velocity, and acceleration (B and C) as a function of target distance. The sampled head movement signal was too noisy to perform jerk analyses. As expected and in line with previous studies, e.g., [12], linear regressions confirmed that the amplitude of head movements (i.e., the range along the principal axis) increased as the simulated distance of the object increased, *R^2^* = .893, slope = 0.255, *p* <. 001 and *R^2^* = .711, slope = 0.105, *p* <. 001, respectively for the Near and Far groups. In addition, the linear regressions performed on the average norm of instantaneous head velocity revealed that participants also increased the speed at which they were moving as the distance of the simulated object increased, *R^2^* = .945, slope = 0.083, *p* <. 001 and *R^2^* = .860, slope = 0.054, *p* <. 001. Similarly, the regressions indicated that the average norm of instantaneous head acceleration increased as the distance of the simulated object increased, *R^2^* = .804, slope = 0.244, *p* <. 001 and *R^2^* = .815, slope = 0.242, *p* <. 001.

### Movements, information, and performance

**Fig 7:**
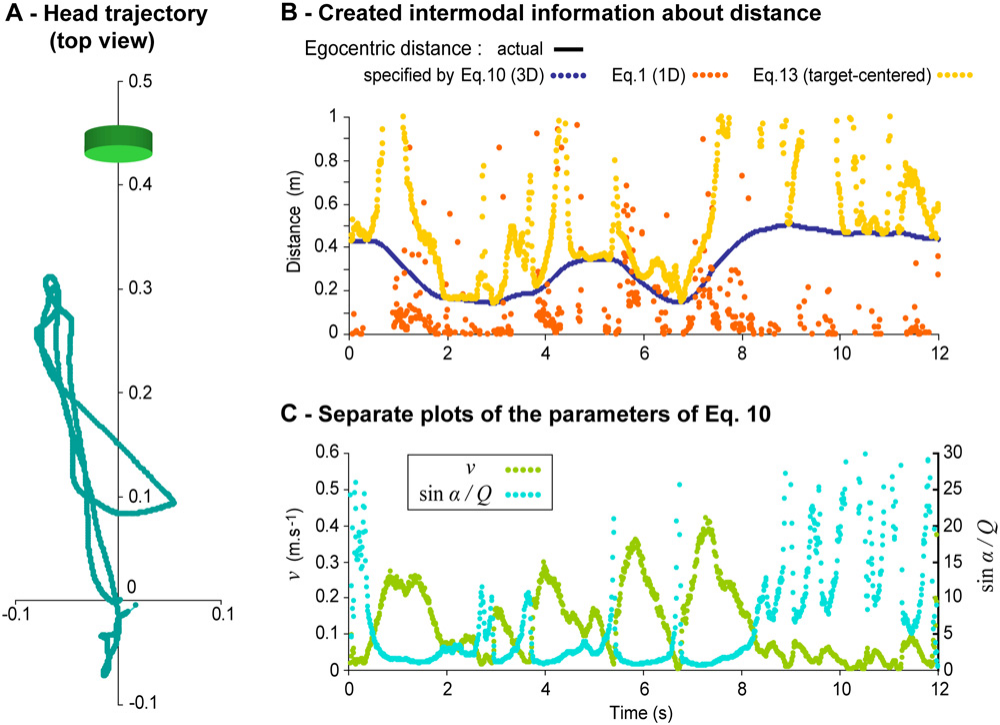
Distance information created through movement during one representative trial in the Movement condition. Data from participant Near 2, trial 33. (A) Head trajectory (bird’s eye view). The scale is in meters. The green cylinder indicates the location and size of the virtual target. (B) Evolution over time of the actual distance of the target and of the distance specified by Eqs 10, 1 and 13. The curve for the actual distance is not visible on the plot because of the perfect overlap with the distance specified by Eq 10. (C) Evolution over time of the optic and inertial components of Ii as formalized in Eq 10.

We also applied our equations to real movement data sampled in the Movement condition for all trials and participants. As expected, Eq 10 (dedicated to 3D movements) provided accurate information about distance 100% of the time. Interestingly, the analyses also revealed that the participants moved such that the simplified equation dedicated to movements orthogonal to the direction of the target (i.e., on a target-centered sphere; 13) specified the actual egocentric distance (±5cm) 28.7% of the time (*CI*_.*95*_ = 22.8, 31.6). In contrast, the equation dedicated to rectilinear movements (Eq 1; e.g., [14,16]) specified the actual egocentric distance only 3.5% of the time (*CI*_.*95*_ = 2.3, 4.0). Fig 7 illustrates these results using data from one participant from the Near group in one representative Movement trial. Head trajectory (seen from above) is plotted on the left side (A). The top graph on the right represents the information about egocentric distance generated by the participant during the trial, as specified by Eqs 10, 1 and 13 (B). The bottom right graph (C) shows the evolution of optic and inertial parameters of *Ii* (Eq 10) over the same period. Taken together, the graph plots B and C underline that distance information is neither available in optics nor in inertia but only in the intermodal relation across the two.

## Discussion

Behavior causes simultaneous, inter-related changes in the structure of multiple forms of ambient energy. These emergent interrelations can constitute higher-order information that differs qualitatively from patterns that exist in individual forms of ambient energy [6]. Previous research has shown that information about an object’s heaviness exists in (and can be perceived from) the relation across optical and inertial consequences of wielding the objet [31]. In this article, we formalized a property of potential sensory stimulation, *Ii*, that is deterministically related to the egocentric distance of objects. *Ii* extends across two forms of ambient energy, optics and inertia. In an experiment, we manipulated the availability of *Ii* independent of the availability of patterns in individual forms of ambient energy, and we investigated its influence on participants’ ability to judge whether a virtual object was within reach.

The results revealed that judgments of reachability were most precise (steeper slopes) and most accurate (lower absolute error) when information about egocentric distance was available through *Ii* (the Movement condition) than when it was not (the Playback and Static conditions). We begin this section by discussing some aspects of the results that, we argue, arise from methodological factors.

### Judgments in the Playback condition

The smooth changes in judgments observed in the Playback condition were unexpected, given the ambiguity of pure optic flow relative to scale. These changes might occur if participants scaled optics using stored or memorized qualities of their previous movements [12]. An alternative interpretation can be derived from considering exploratory movements and their consequences on optic flow. Theoretically a given optic flow can correspond to an infinite number of combinations of target distance and perceiver movement. In practice, however, constraints proper to the animal (e.g., biomechanical), the environment (e.g., gravity) or the task (e.g., remaining seated), reduce this infinity to a smaller set of ecologically valid combinations [32]. In our experiment, participants increased their movement amplitude, speed, acceleration, and jerk as the depicted distance of the virtual target increased across trials. However, this regulation did not fully compensate for the concurrent increase in the distance at which the target was depicted. As a result, the average speed, acceleration and jerk at which the direction of the target changed relative to the perceiver (i.e., in optic flow) decreased as a function of target distance (see S2 Fig). Thus, pure optic flow provided information about the relative distance of targets across trials. The low magnitude of curves’ slopes in the Playback condition suggests that—in this condition—participants might have sorted targets in terms of relative distance (i.e., closer vs. farther), rather than according to their scaled distance. In any event, given that optic flow was identical in the Playback and Movement conditions, the better performance that we observed in the Movement condition can be explained only in terms of the presence and use of patterns in ambient arrays above and beyond optic flow.

### Influence of centering bias on judgments

The accuracy of judgments appeared to be affected by a so-called centering bias, e.g., [27,28]. By separating participants’ actual maximum reachable distance (MR_A_) from the middle of the set of depicted distances, we were able to assess independently the influence of each of these factors. Such independent control of the investigated variable (i.e., MR_A_) and methodological factors (i.e., the symmetry of the range of targets around MR_A_) is rare in psychophysical experiments, e.g., [12,13,27]. Thus, some of the effects observed in previous studies may reflect confounding of these factors. Here in our Movement condition, despite the concurrent influence of the bias, the perceived maximum reachable distance (MR_P_) was consistently attracted toward the actual boundary (MR_A_), confirming that scaled distance was perceived. Overall, the constant error in that condition was only 6.5 cm in average, replicating the overestimation of the distance perceived reachable in real environments reported in several studies, e.g., [19,33–35] and the additional compression of distances generally observed in virtual environments, e.g., [36,37].

### Exploratory movements provide higher-order information about egocentric distance

Movement facilitates perception. In our experiment, movements were necessary to generate emergent, higher order patterns that provided unambiguous information about egocentric distance. The formal analysis of these patterns underlined the fact that the information that is available to a perceiver is contingent on the characteristics of his/her exploratory movement (see Analysis of Available Information). Our participants were free to move their eyes, head, and torso. We argue that it is for this reason that the present study provides the first evidence that exploratory activity can be sufficient for the perception of egocentric distance.

Previous studies investigating egocentric distance perception from optic flow have restricted participants to one-dimensional oscillatory movements in a specific direction [12,13,18–25]. With a few exceptions [25], they focused on two directions: toward the object or laterally. Generally this restriction was adopted because these directions were assumed to generate two different optical patterns (radial flow and motion parallax, respectively). By contrast, in ordinary life a forward head movement always entails a downward movement and thus generates motion parallax (up/down) in addition to radial optic flow [22]. A similar analysis also applies to side-to-side head movements, which, by modifying head-object distance, also generate radial flow patterns in addition to motion parallax (only a curvilinear movement orthogonal to the direction of the object, can generate pure motion parallax). When distance perception was compared across these two types of movement in those earlier studies, participants exhibited better distance estimates when moving toward the target than when moving laterally [13,22]. In our experiment, the majority of principal directions about which movements were organized (85.6%) were localized in the hemifield centered on the direction of the target (i.e., [-45°, +45°] in azimuth). However, in agreement with our model, which does not suggest any advantage of moving in a particular absolute direction (e.g., Eqs 1, 1, 4–5, 7,10–12,13–15; see also [14,16]), within this hemifield participants did not favor a unique direction of movement. Rather, the principal direction varied across participants and, to a smaller extent, across trials for individual participants.

Our equations do not simplify for any particular absolute direction of movement; however, they undergo a qualitative change when the angle *α* between heading (i.e., the direction of movement) and the direction of the target is kept constant. For example, when the point of observation follows a curvilinear path at constant distance from the target, specified distance no longer depends upon the instantaneous direction of the target relative to heading (Eqs 13–15). Such simplified patterns could turn out to be particularly useful in presence of a visual scene that lacks rich optical structure, such as when a single object is presented in an otherwise empty visual field (as in many experiments, including ours). Indeed, this impoverished optical structure reduces available information about heading and impairs its perception by humans [38], thereby limiting available information about object-relative heading. In our experiment, approximately 30% of the time participants generated accurate distance information through movements that were orthogonal to the direction of the object (Eqs 13–15). This tendency varied among participants. Participants that were more likely to move in this way (i.e., generating information through these simplified equations) tended to exhibit greater accuracy in their reachability judgments (see S2 Text for details). Thus, these participants may have exploited these simplified patterns as a means to ease or improve the generation and/or pickup of information.

Individuals with only one eye have been observed to utilize larger and more rapid head movements than persons for whom monocular vision is temporary [39]. Before jumping over a gap, Mongolian gerbils move their head with amplitude and velocity that are correlated to the size of the gap [40]. Our experiment extended this finding to humans. Participants increased the amplitude, speed, and acceleration of their head movements for more distant targets. By doing so, participants tended to minimize the decrease in the (angular) amplitude, speed, and acceleration at which the direction of the object changed relative to their head (i.e., the optical parameters in Eqs 1–15). Interestingly, if participants effectively attempted to preserve a certain amount of optical kinematics, the need to increase the head kinematics as a function of target’s distance was probably heightened by our experimental apparatus, which altered the spatiotemporal resolution of optic flow. In particular, the angular resolution of optic flow was constrained by the size of pixels on the HMD screen, the distance of the screen relative to the eye and the lens that lied in between.

Altogether, our results suggest that participants engaged in types of exploratory activity that would tend to increase or preserve the salience of *Ii*. Interestingly, this shaping of exploratory behavior by informational constraints occurred despite the fact that participants had no training with our virtual environment and were not given any practice trials or feedback about their performance. Within this perspective, perception influenced action, which influenced perception, in a continuous process that unfolded until the desired outcome was achieved (e.g., information about egocentric distance; cf. [41,42]). In a similar fashion, infants as early as 5 to 12 weeks old have been observed to increase their sucking rate when the clarity (i.e., focus) of the visual scene is made contingent on sucking rate [43], see also [44]. In adults, it has been proposed that optical information could serve to enhance and facilitate the detection of rotational inertia, e.g., [31,45].

### Conclusion

By themselves, the optical consequences of a perceiver’s movement are not sufficient to specify egocentric distance [11,15,17,46–48]. Information about egocentric distance requires that optic flow be scaled. In our experiment, optic flow might have been scaled as a fact of ambient energy through a higher order pattern comprising haptic/gravito-inertial stimulation, as we suggest. Alternatively, optic flow might have been scaled via an efferent copy of motor commands. The empirical findings of the present study cannot resolve this debate. Whatever the interpretation may be, information about egocentric distance that exists in *Ii* has at least two important characteristics. First, this information is intermodal in a novel sense: The information exists as a pattern in ambient energy prior to stimulation of any receptor systems. The distance information that participants exploited is not available in any of its constitutive modal patterns (e.g., those available separately to the visual, kinaesthetic, and vestibular systems), but only in a higher order pattern that extends across these forms of ambient energy (see Fig 7). Second, this information is generated by the animal through its exploratory activity [7]. By moving, a perceiver reveals higher order, invariant structures that are the consequences of his/her movement, and these structures specify the dynamics of the perceiver-environment system that gives rise to them. The emergent, higher order relations across different forms of ambient energy are not due to chance [6,49]. For example, there is a tripartite causal relation between changes in head position relative to the environment (which structure haptic/gravito-inertial stimulation), changes in objects angular direction relative to the head (which structure optic flow) and changes in head-object distance. As a consequence, the higher order invariant pattern specifying egocentric distance is a parameter that is available to the animal as a consequence of its movement. It does not need to be inferred. Altogether, this underlines that there is more than spatio-temporal redundancy or coherence across the dimensions of stimulation (optics, acoustics, haptics, etc.): In addition, there is information. Our results suggest that this emergent, higher order information is sufficient for perception of egocentric distance and that humans are sensitive to this information and can use it to perceive egocentric distance.

## Materials and Methods

### Participants

Thirteen undergraduate students from the University of Minnesota and one of the authors (6 females, 8 males) volunteered to participate in the experiment. Students received academic credits for their participation. Participants ranged in age from 18 to 42 years (*M* = 23, *SD* = 5.6), and in height from 155 to 193 cm (*M* = 172.9, *SD* = 11.6). Their arm length, measured from the acromion to the tip of the index finger ranged from 61 to 81 cm (*M* = 72.4, *SD* = 5.6). All participants had normal or corrected-to-normal vision and all but one were naïve to the purpose of the experiment.

### Experimental task

Our response measure differed from that used in some previous studies. Some studies, e.g., [12,13,24] required participants to report their percepts using numerical scales (e.g., in inches, “between 50 and 100 cm”). Such responses are problematic because they confound perceptual accuracy with the participants’ ability to convert perception into numbers [19,34,36]. Following other studies, e.g., [19,50] we used a response measure that did not require such a conversion.

Previous studies have shown that adults exhibit refined knowledge of whether an object is within reach, and even of the particular type of reaching (arm-only, leaning at the waist, taking a step, and so on) that will be optimal to reach a given object at a given distance [33,35,51]. Even infants can do this: Yonas and Hartman [52] found that 5-months old infants regularly reached toward objects that were less than one arm length distant, but did not reach for objects that were more than one arm length away. In this work, we focus on the patterns within stimulation that could support such perception. Presumably, the prospective perception of whether an object is within reach requires relational information about the egocentric distance of the object and the distance that can be reached by the actor/perceiver (which depends on arm length, posture, tool characteristics, etc.; e.g., [53–55]). We do not claim (nor disclaim) that egocentric distance information is relevant for the *online* regulation of reaching movements. Our concern is with affordance perception, the role of which is to support the *prospective* control of actions (e.g., movement selection). We hypothesize that the intermodal pattern we identified and formalized can provide information about the former aspect (i.e., the distance of the targeted object) and thus that manipulating this information would influence participants’ perception of whether the object is within reach. Participants were asked to judge verbally (yes/no) whether they could reach a stationary virtual target, whose distance was varied among trials. In addition, they were asked to rank verbally how confident they were about their reachability judgments, using a 1–5 scale with 5 being the most confident. “Reaching”, meant “reaching with your finger while extending your preferred arm, without twisting shoulders or leaning forward”. Our definition of reaching corresponded to a 1 degree of freedom reach in the terminology used in previous studies [33,35].

### Apparatus

The set up is illustrated in Fig 2A. Participants were seated on a height-adjustable office chair, in a dark room. They were presented with stationary virtual objects at eye height through an HMD (Visette Pro, Cyberminds, Netherlands). Among the two LCD matrixes of the HMD (640 × 480 pixels each, 60 Hz), only that facing the (self-reported) preferred eye was turned on, yielding a monocular field of 60° × 46°. The simulated object was a green and blue coin-like cylinder displayed in an otherwise empty (black) visual field (Fig 2C). Before each trial, the size of the target was determined as a function of its distance such that its angular size was always 9° at the beginning of the trial (the thickness of the target also was set proportionally). To depict a stationary virtual object at a given distance beyond the HMD screen, the participant’s position and orientation were sampled with a 6-dof electromagnetic sensor (Flock of Birds, Ascension, Burlington, VT, USA) and used to drive in real time the display of the object on the screen of the HMD (Fig 2B1; S1 Video). The sensor was attached to the HMD, above the eyes, centered (Fig 2A). Although improved in recent decades, e.g. [56], the principle used to depict a virtual object at a distance traces back to at least to Wallace [57]. With our apparatus, many visual cues to distance were absent, and most others were neutralized (i.e., they were influenced by the eye’s distance relative to the screen and not to the virtual object). The invariant pattern across inertial and optical consequences of participant’ movement was the only remaining source of information about the egocentric distance of the simulated target. We were able to manipulate that pattern, either by reproducing the relation existing in real world, or by simply breaking it. A second electromagnetic sensor was used (located on a real target) for the preliminary measurement of the actual maximal reachable distance (MR_A_; see below).

### Design and procedure

All participants gave their written informed consent to participate in the study. The protocol was approved by the Institutional Review Board of the University of Minnesota where the data were collected. Before the beginning of the experiment, we measured for each participant the maximal distance from which they could actually reach an object (MR_A_), by having them performing real reaching actions (with their arm only, see Task section above). The MR_A_ was measured from the preferred eye to the target, using the method of limits [58–60]. Participants were presented real targets at eye level and asked to reach them using their arm only. After each reach, the target was pulled back centimeter-by-centimeter (measured with the electromagnetic sensors) to increase the egocentric distance. This procedure was repeated until the target was too far to be reached for two consecutive trials. The operation was then restarted with decreasing distances, starting from unreachable targets and continuing until the participant could reach two consecutive targets. MR_A_ was defined as the mean of the last and first distances judged reachable in respectively these ascending and descending series. The obtained values for MR_A_ ranged from 50 to 72 cm (*M* = 62.1, *SD* = 5.3).

The judgment session involved three experimental conditions (Fig 2B) in which we manipulated the availability of the different components of the invariant relation specifying egocentric distance (i.e., in Eqs. 1, 4, 10–12, 13–15). In the Movement condition, participants were encouraged to freely explore the scene by moving their head relative to the virtual object prior to giving their judgment. The closed-loop updating system was activated, such that the information described in Eq 10 (and related forms) was available when the perceiver moved. Accordingly, we expected that participants would give precise and accurate reachability judgments. To appreciate performance in that main experimental condition, we designed two control conditions. In the Stationary condition, participants were instructed not to move while looking at the target. They were not physically restrained but the closed-loop updating display was deactivated, such that changes in haptic/gravito-inertial stimulation were minimized and not related to the display of the object. In the Playback condition, participants were also instructed to remain still, but they were presented a moving display of the target. The movements of the displayed target were driven by previously recorded movements of the same participant played back from earlier trials (see S1 Video). Thus, the Playback condition provided optical stimulation that was driven by body movement, but the relation between head movements and optics was open loop [12,13,56,61].

Each judgment followed the same procedure. Participants opened their eyes on a “Go” signal, took as much time as they wished and then said “yes” or “no” to indicate whether they judged the virtual target to be within reach, followed by a number between 1 and 5 for the confidence rating. As soon as they gave their responses, the simulation was turned off (dark screen) and participants were asked to close their eyes until the beginning of the next trial.

Targets were presented at 17 different distances, calculated for each participant on the basis of his/her MR_A_. Participants were randomly split into Near and Far groups. For the Near Group, target distances ranged from 23% to 135% of each participant’s MR_A_, while they ranged from 72 to 184% for the Far Group. The motivation for having two groups was to control for potential centering bias, that is, a potential influence on perceived maximum reachable distance, MR_P_, of the localization of MR_A_ within the tested interval [27,28]. There were five trials at each distance. All trials were randomized within condition, then grouped by blocks of 17 trials of the same condition. All blocks were then randomized, with the only restriction that at least one block of Movement trials be present before the first block of Playback trials, in order to feed the database of recorded head movements. Participants performed a total of 255 trials (15 blocks of 17 trials), which were divided in two sessions of 45 to 60 minutes each, conducted on different days.

### Analysis of judgment data

In psychophysical experiments, a typical response curve exhibits a clear transition from a majority of “yes” (e.g., reachable) to a majority of “no” (e.g., not reachable) judgments as the stimulus (e.g., simulated distance) increases or decreases. Ideally, transitions are located around an expected threshold. To analyze this transition, we fitted psychometric functions to the percentage of positive responses expressed as a function of the object’s distance [59,62–64] using the psignifit toolbox (version 2.5.6 for Matlab http://bootstrap-software.org/psignifit/):

$$\Psi \;\left(x,\theta ,\pi_{c},\pi_{l}\right)=\pi_{c}+\left(1-\pi_{c}-\pi_{l}\right)\;F\left(x,\theta \right)$$ (16)

where the probability *Ψ* that the participant gives a positive answer is defined as a function of the probability *π*_*l*_ that she/he answers independently of the stimulus intensity *x* (*miss rate*), the stimulus-independent probability (or *guess rate*) *π*_*c*_ that she/he gives a positive response, and the probability *F*(*x*, θ) of positively evaluating the stimulus. Following Wichmann and Hill, we did not fix the values of π_*l*_ and π_*c*_ *a priori*. Rather, we constrained their values to lie within a reasonable interval (i.e., [0, 0.05]). In accordance with previous work on affordance judgments, e.g., [58,65], we used a logistic function for *F*. We derived two indicators of performance from *F*: the abscissa of the point where positive and negative judgments were balanced and the value of the slope at this point. The first provided a measure of the perceived maximum reachable distance (MR_P_) and was used to quantify judgment accuracy through absolute error. The second indicator was our measure of the precision of judgments (i.e., consistency). Goodness-of-fit was assessed with the deviance test based on Monte-Carlo simulations described in Wichmann and Hill [63] and confidence interval of each parameters were found by the BCa bootstrap method implemented by psignifit, based on 4000 simulations [64]. Altogether, these psychophysical tools permitted a precise analysis of the accuracy and precision of judgments, as well as control over the relevance and consistency of these dependent variables. We modelled the data for each participant in each condition (individual fits) and for all participants in each condition (global fits). We used results from individual fits to compare experimental conditions, while global fits provided good descriptions of the results at group level.

### Analysis of movement data

To attenuate the noise of the sensor, movement data were filtered using a two-way low-pass Butterworth filter (4 order, 12 Hz cutoff frequency). From the filtered data we computed for each trial the principal direction of movement, the amplitude of movement (range) along this preferred axis, and the average norm of instantaneous velocity, acceleration and jerk. The principal direction of movement was computed from the distribution of instantaneous directions of movement by taking the eigenvector corresponding to the largest eigenvalue of the orientation matrix (also called scatter matrix when normalized by sample size; cf. [66], p.162, and [67], p.233). The eigenvalues provided a measure of the proportion of variance in movement direction explained by each of the eigenvectors.

Additionally, we analyzed exploratory movements in the light of available intermodal information as we formalized it through Eq 10 and related forms. Using head movement data, we calculated for each participant the instantaneous egocentric distances that were specified at each point of the trajectory by Eq 10 dedicated to 3D movements, by Eq 1 dedicated to 1D movements, and by Eq 13 dedicated to movements orthogonal to the direction of the target (e.g., tangential to a target-centered sphere). Using these, for each equation we computed the percentage of points (i.e., the amount of time) for which the difference between specified and actual distance was lower than 5 cm. This provided a measure of the amount of time during which each equation provided accurate information about distance (given each participant’s exploratory motion).

### Inferential statistics

Except where otherwise indicated, we used an alpha level of. 05 for all inferential tests. We also report effect sizes for each test using partial η^2^ for ANOVA (noted pη^2^) and Cliff’s *dw* and *d* statistic for nonparametric tests [68]. In repeated measures designs, the *dw* statistic is the proportion of participants who change in one direction minus the proportion who change in the opposite direction. For independent samples, the *d* statistic indicates the proportion of scores from one population that are higher than those from the other, minus the reverse proportion [68], p.495. *dw* and *d* vary from 1 (all scores greater at the second test or in the second population) to -1 (all scores smaller at the second test/population).

## Supporting Information

S1 FigOptical and non optical consequences of a 3D movement relative to a stationary object.(A) Using a Cartesian coordinate system, the egocentric distance can be expressed as a function of directional parameters (*α*, *θ*) describing the motion of the point of observation in the plane defined by *O* and $\vec{v}$ (a), two more directional parameters *φ* and *ψ* (b, c) characterizing the orientation of that plane relative to an earth-fixed reference frame $\left({\vec{x}}_{0},{\vec{y}}_{0},{\vec{z}}_{0}\right)$, and linear parameters about head movements (*v*). (B) Using a spherical coordinate system, the egocentric distance can be expressed as a function of directional parameters (Φ, *δ*) and linear parameters about head movements (*v*) (see S1 Text for details).(PDF)

S2 Fig Kinematics of the direction of the object relative to the point of observation. Optical parameters in Eqs 10–11: (A) Parameter *Q* in deg.s^-1^. (B) Parameter *Q* in deg.s^-2^. In the two panels, average instantaneous values are plotted as a function of the distance at which the target was simulated at the beginning of the trial (expressed as a proportion of the actual maximum reachable distance MR_A_).
(PDF)

S1 Table Perceived maximum reachable distance (MR_P_) and slope derived from judgments curves. The mean values (*M*) and confidence interval (*CI*_.*95*_) of MR_P_ and slope are calculated from significant individual fits in each experimental condition (Movement, Stationary, Playback). The *N* values indicate the number of participants included in the analyses (i.e., whom deviance test assessing goodness of fit was significant.
(PDF)

S2 Table Second order polynomial regressions fitted to confidence ratings. The values obtained for each parameter of the equation (*Ax^2^ + Bx + C*), the determination coefficient of the fit (*R^2^*) and the *p* statistics associated to each parameter of the regression are shown for each group of participant in each experimental condition.
(PDF)

S1 Text Mathematical derivation of Eqs 5 and 7. Eqs 5 and 7. These equations describe the information about scaled egocentric distance available in the intermodal consequences of 3D movements, using Cartesian and spherical coordinates, respectively.
(PDF)

S2 Text Influence of the amount of information generated on performance. Comparison of the accuracy of reachability judgments between participants who (according to Eq 13) most and least often generated accurate information about distance by moving orthogonal to the direction of the target.
(PDF)

S1 Video Video caption showing what was displayed on the screen of the HMD wore by participants. The display of the object is driven by previously recorded movements played back from one representative trial of the Movement condition (participant Far 1, trial 12).
(MOV)
