# Supplementary material for: Exploratory Movement Generates Higher-Order Information That Is Sufficient for Accurate Perception of Scaled Egocentric Distance
Source: PLoS One. 2015 Apr 9;10(4):e0120025. doi: 10.1371/journal.pone.0120025 (PMC4391914; doi:10.1371/journal.pone.0120025)
Supplement: S1 Fig — (A) Using a Cartesian coordinate system, the egocentric distance can be expressed as a function of directional parameters (α, θ) describing the motion of the point of observation in the plane defined by O and v→ (a), two more directional parameters φ and ψ (b, c) characterizing the orientation of that plane relative to an earth-fixed reference frame (x→0,y→0,z→0), and linear parameters about head movements (v). (B) Using a spherical coordinate system, the egocentric distance can be expressed as a function of directional parameters (Φ, δ) and linear parameters about head movements (v) (see S1 Text for details). (PDF) [file pone.0120025.s001.pdf]

Exploratory movement generates higher-order information that is sufficient for accurate perception of scaled egocentric distance

Bruno Mantel, Thomas A. Stoffregen, Alain Campbell, Benoît G. Bardy

Supporting Information

Figure S1

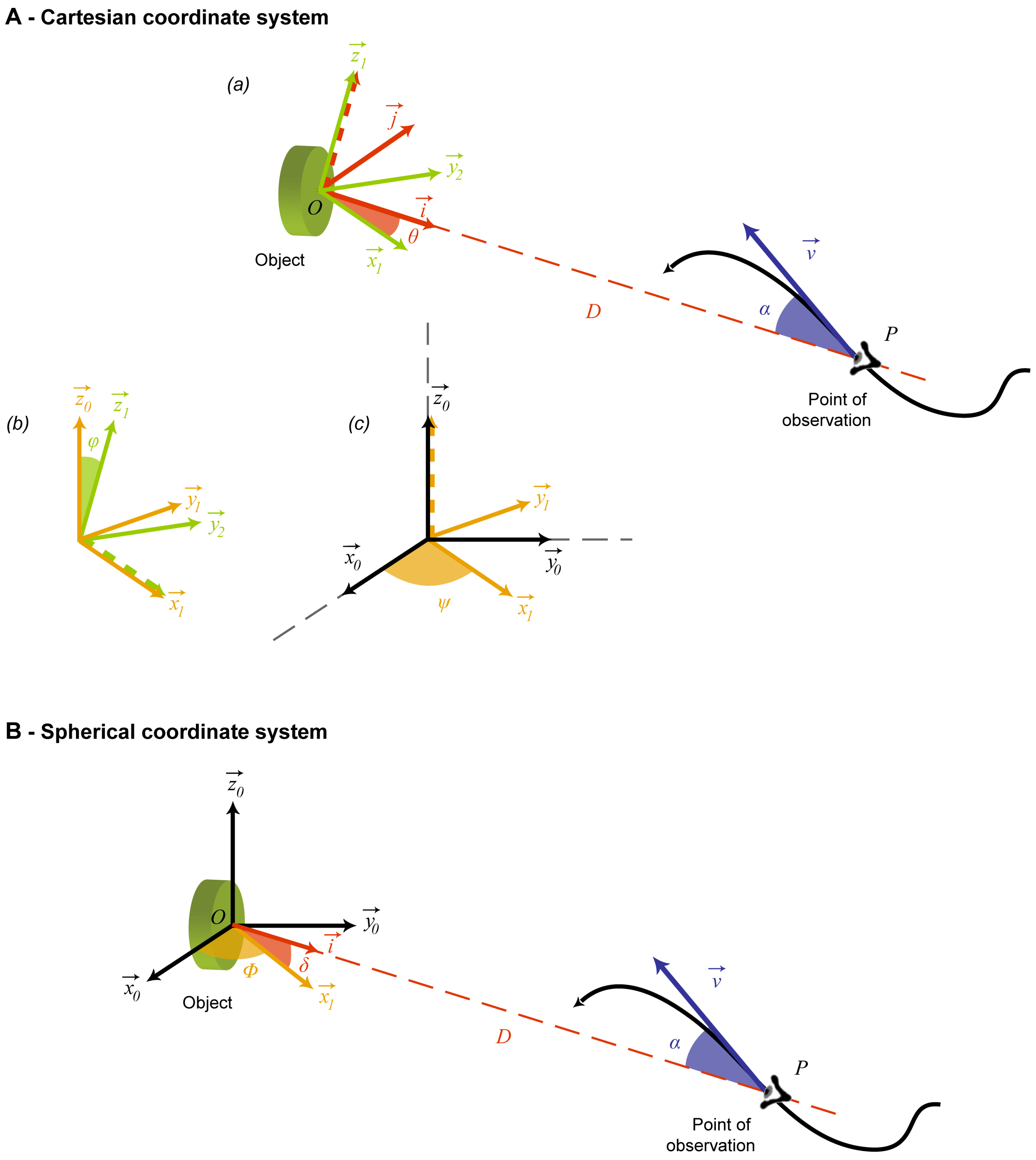

Figure S1. Optical and non optical consequences of a 3D movement relative to a stationary object. (A) Using a Cartesian coordinate system, the egocentric distance can be expressed as a function of directional parameters (α, θ) describing the motion of the point of observation in the plane defined by O and $\vec{v}$ (a), two more directional parameters φ and ψ (b, c) characterizing the orientation of that plane relative to an earth-fixed reference frame ($\vec{x_0}, \vec{y_0}, \vec{z_0}$) and linear parameters about head movements (v). (B) Using a spherical coordinate system, the egocentric distance can be expressed as a function of directional parameters (Φ, δ) and linear parameters about head movements (v). (see Text S1 for details).
