# Supplementary material for: Exploratory Movement Generates Higher-Order Information That Is Sufficient for Accurate Perception of Scaled Egocentric Distance
Source: PLoS One. 2015 Apr 9;10(4):e0120025. doi: 10.1371/journal.pone.0120025 (PMC4391914; doi:10.1371/journal.pone.0120025)
Supplement: S2 Fig — Optical parameters in Eqs 10–11: (A) Parameter Q in deg.s-1. (B) Parameter Q in deg.s-2. In the two panels, average instantaneous values are plotted as a function of the distance at which the target was simulated at the beginning of the trial (expressed as a proportion of the actual maximum reachable distance MRA). (PDF) [file pone.0120025.s002.pdf]

Exploratory movement generates higher-order information that is sufficient for accurate perception of scaled egocentric distance

Bruno Mantel, Thomas A. Stoffregen, Alain Campbell, Benoît G. Bardy

Supporting Information

Figure S2

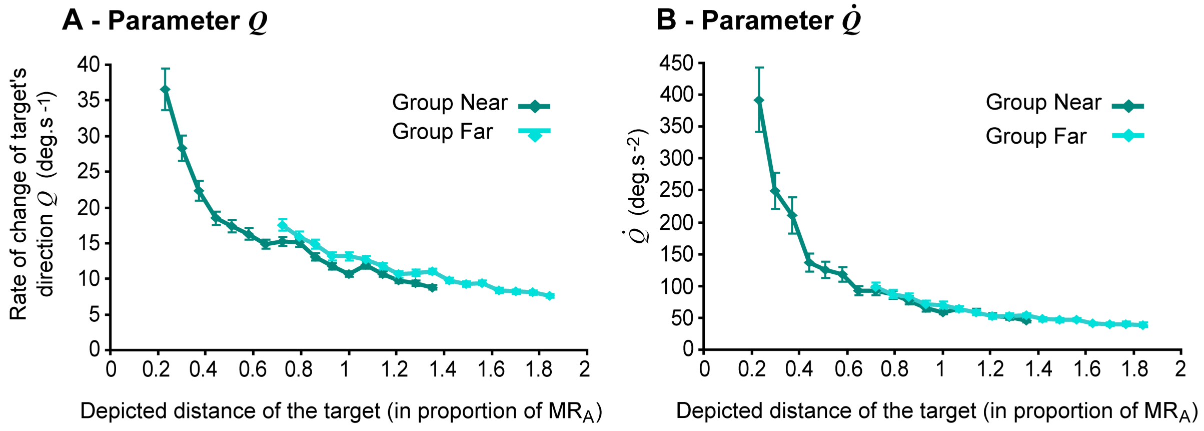

Figure S2. Kinematics of the direction of the object relative to the point of observation (optical parameters in Eq. 10-11). (A) Parameter Q in deg.s^{-1}. (B) Parameter $\dot{Q}$ in deg.s^{-2}. In the two panels, average instantaneous values are plotted as a function of the distance at which the target was simulated at the beginning of the trial (expressed as a proportion of the actual maximum reachable distance MR_A).
