# Supplementary material for: Exploratory Movement Generates Higher-Order Information That Is Sufficient for Accurate Perception of Scaled Egocentric Distance
Source: PLoS One. 2015 Apr 9;10(4):e0120025. doi: 10.1371/journal.pone.0120025 (PMC4391914; doi:10.1371/journal.pone.0120025)
Supplement: S1 Table — The mean values (M) and confidence interval (CI .95) of MRP and slope are calculated from significant individual fits in each experimental condition (Movement, Stationary, Playback). The N values indicate the number of participants included in the analyses (i.e., whom deviance test assessing goodness of fit was significant. (PDF) [file pone.0120025.s003.pdf]

Exploratory movement generates higher-order information that is sufficient for accurate perception of scaled egocentric distance

Bruno Mantel, Thomas A. Stoffregen, Alain Campbell, Benoît G. Bardy

Supporting Information

Table S1

Table S1. Perceived maximum reachable distance (MR_P) and slope derived from judgments curves.

		MR_P		Slope	
**Condition**	N	M	$CI_{.95}$	M	$CI_{.95}$
**Movement**	13	1.11	0.98, 1.24	-2.7	-3.6, -1.8
**Stationary**	7	1.28	0.24, 2.32	-0.5	-1.2, 0.3
**Playback**	14	2.08	0.83, 3.32	-0.5	-0.7, -0.4

The mean values (M) and confidence interval ($CI_{.95}$) of MR_P and slope are calculated from significant individual fits in each experimental condition (Movement, Stationary, Playback). The N values indicate the number of participants included in the analyses (i.e., whom deviance test assessing goodness of fit test was significant.
