# Supplementary material for: Exploratory Movement Generates Higher-Order Information That Is Sufficient for Accurate Perception of Scaled Egocentric Distance
Source: PLoS One. 2015 Apr 9;10(4):e0120025. doi: 10.1371/journal.pone.0120025 (PMC4391914; doi:10.1371/journal.pone.0120025)
Supplement: S2 Table — The values obtained for each parameter of the equation (Ax2 + Bx + C), the determination coefficient of the fit (R2) and the p statistics associated to each parameter of the regression are shown for each group of participant in each experimental condition. (PDF) [file pone.0120025.s004.pdf]

Exploratory movement generates higher-order information that is sufficient for accurate perception of scaled egocentric distance

Bruno Mantel, Thomas A. Stoffregen, Alain Campbell, Benoît G. Bardy

Supporting Information

Table S2

Table S2. Second order polynomial regressions fitted to confidence ratings.

Condition	Group	Parameters values *A*	*B*	*C*	Fit R^2	*p* statistics *A*	*B*	*C*
**Movement**	**Near**	3.18	-6.05	6.47	.883	$< .001$	$< .001$	$< .001$
	**Far**	2.15	-5.95	7.15	.855	$< .001$	$< .001$	$< .001$
**Stationary**	**Near**	0.50	-0.83	2.49	.125	*ns*	*ns*	$< .001$
	**Far**	-0.49	1.29	1.04	.164	*ns*	*ns*	$< .05$
**Playback**	**Near**	-0.08	-0.85	4.65	.821	*ns*	*ns*	$< .001$
	**Far**	0.38	-1.54	4.06	.633	*ns*	*ns*	$< .001$

The values obtained for each parameter of the equation ($Ax^2 + Bx + C$), the determination coefficient of the fit (R^2) and the p statistics associated to each parameter of the regression are shown for each group of participant in each experimental condition.
