# Supplementary material for: Exploratory Movement Generates Higher-Order Information That Is Sufficient for Accurate Perception of Scaled Egocentric Distance
Source: PLoS One. 2015 Apr 9;10(4):e0120025. doi: 10.1371/journal.pone.0120025 (PMC4391914; doi:10.1371/journal.pone.0120025)
Supplement: S1 Text — These equations describe the information about scaled egocentric distance available in the intermodal consequences of 3D movements, using Cartesian and spherical coordinates, respectively. (PDF) [file pone.0120025.s005.pdf]

Exploratory movement generates higher-order information that is sufficient for accurate perception of scaled egocentric distance

Bruno Mantel, Thomas A. Stoffregen, Alain Campbell, Benoît G. Bardy

Supporting Information

Text S1

A. Cartesian coordinate system

From the three following change of basis matrixes,

$$\begin{array}{c|ccc} & \overrightarrow{x_1} & \overrightarrow{y_1} & \overrightarrow{z_0} \\ \overrightarrow{x_0} & cos\psi & -sin\psi & \\ \overrightarrow{y_0} & sin\psi & cos\psi & \\ \overrightarrow{z_0} & & & \end{array} \qquad \begin{array}{c|ccc} & \overrightarrow{x_1} & \overrightarrow{y_2} & \overrightarrow{z_1} \\ \overrightarrow{x_1} & & & \\ \overrightarrow{y_1} & & cos\varphi & -sin\varphi \\ \overrightarrow{z_0} & & sin\varphi & cos\varphi \end{array} \qquad \begin{array}{c|ccc} & \overrightarrow{i} & \overrightarrow{j} & \overrightarrow{z_1} \\ \overrightarrow{x_1} & cos\theta & -sin\theta & \\ \overrightarrow{y_2} & sin\theta & cos\theta & \\ \overrightarrow{z_1} & & & \end{array}$$

we have:

$$\overrightarrow{i} = cos\theta\,\overrightarrow{x_1} + sin\theta\,\overrightarrow{y_2}$$

and

$$\frac{d\,\overrightarrow{i}}{dt} = \dot{\theta}\,\overrightarrow{j} + cos\theta\frac{d\overrightarrow{x_1}}{dt} + sin\theta\frac{d\overrightarrow{y_2}}{dt}$$

where

$$\frac{d\overrightarrow{x_1}}{dt} = \dot{\psi}\,\overrightarrow{y_1}, \qquad \frac{d\overrightarrow{y_2}}{dt} = \dot{\varphi}\,\overrightarrow{z_1} + cos\varphi\frac{d\overrightarrow{y_1}}{dt} \qquad \text{and} \qquad \frac{d\overrightarrow{y_1}}{dt} = -\dot{\psi}\,\overrightarrow{x_1}$$

and therefore:

$$\frac{d\,\overrightarrow{i}}{dt} = \dot{\theta}\,\overrightarrow{j} + \dot{\psi}\,cos\theta\,\overrightarrow{y_1} + sin\theta\left(\dot{\varphi}\,\overrightarrow{z_1} - \dot{\psi}\,cos\varphi\,\overrightarrow{x_1}\right) \tag{S1}$$

We also know that:

$$\overrightarrow{v} = v\left(cos\alpha\,\overrightarrow{i} + sin\alpha\,\overrightarrow{j}\right) \tag{S2}$$

and that

$$\overrightarrow{v} = \frac{d\overrightarrow{OP}}{dt} = \frac{dD\,\overrightarrow{i}}{dt} = \dot{D}\,\overrightarrow{i} + D\frac{d\,\overrightarrow{i}}{dt} \tag{S3}$$

Thus from Eq. S1-S3 we can write:

$$v\left(cos\alpha\,\overrightarrow{i} + sin\alpha\,\overrightarrow{j}\right) = \dot{D}\,\overrightarrow{i} + D\left(\dot{\theta}\,\overrightarrow{j} + \dot{\psi}\,cos\theta\,\overrightarrow{y_1} + sin\theta\left(\dot{\varphi}\,\overrightarrow{z_1} - \dot{\psi}\,cos\varphi\,\overrightarrow{x_1}\right)\right) \tag{S4}$$

By projecting Eq. S4 on $\overrightarrow{i}$ we obtain:

$$\dot{D} = v\,cos\alpha \tag{S5}$$

which might be useful for the online control of movement, but this is below the scope of the present paper.

By projecting Eq. S4 on $\overrightarrow{j}$ we obtain:

$$D = \frac{v\,sin\alpha}{\dot{\theta} + \dot{\psi}\,cos\varphi} \tag{S6}$$

Since $\overrightarrow{v}$ is in the plane $\left(\overrightarrow{i}, \overrightarrow{j}\right)$, $\frac{d\overrightarrow{j}}{dt}$ is orthogonal to $\overrightarrow{z_1}$ and thus by projecting $\frac{d\,\overrightarrow{i}}{dt}$ (Eq. S1) on $\overrightarrow{z_1}$ we obtain:

$$-\dot{\psi}\,cos\theta\,sin\varphi + \dot{\varphi}\,sin\theta = 0 \tag{S7}$$

indicating that the three parameters are related.

Thus Eq. S6 can be rewritten:

$$D = \frac{v\, sin\alpha}{\dot{\theta} + \dot{\varphi}\frac{sin\theta\, cos\varphi}{cos\theta\, sin\varphi}} = \frac{v\, sin\alpha}{\dot{\theta} + \dot{\varphi}\frac{tan\theta}{tan\varphi}} \tag{S8}$$

B. Spherical coordinate system

The unit vector $\overrightarrow{i}$ is defined as:

$$\overrightarrow{i} = cos\delta\, \overrightarrow{x_1} + sin\delta\, \overrightarrow{z_0}$$

and therefore its first order time derivate is:

$$\frac{d\overrightarrow{i}}{dt} = -\dot{\delta}\, sin\delta\, \overrightarrow{x_1} + \dot{\delta}\, cos\delta\, \overrightarrow{z_0} + cos\delta\frac{d\overrightarrow{x_1}}{dt}$$

where

$$\frac{d\overrightarrow{x_1}}{dt} = -\dot{\Phi}\, sin\Phi\, \overrightarrow{x_0} + \dot{\Phi}\, cos\Phi\, \overrightarrow{y_0} = \dot{\Phi}\, \overrightarrow{y_1}$$

and thus

$$\frac{d\overrightarrow{i}}{dt} = -\dot{\delta}\, sin\delta\, \overrightarrow{x_1} + \dot{\Phi}\, cos\delta\, \overrightarrow{y_1} + \dot{\delta} cos\delta \overrightarrow{z_0} \tag{S9}$$

By definition of the cross product, we have:

$$\left\| \overrightarrow{i} \wedge \overrightarrow{v} \right\| = v|sin\alpha| \tag{S10}$$

From Eq. S3, we also know that:

$$\left\| \overrightarrow{i} \wedge \overrightarrow{v} \right\| = \left\| \overrightarrow{i} \wedge (\dot{D}\overrightarrow{i} + D\frac{d\overrightarrow{i}}{dt}) \right\| = \left\| \overrightarrow{i} \wedge D\frac{d\overrightarrow{i}}{dt} \right\| \tag{S11}$$

and thus

$$v\, sin\alpha = D\left\| \overrightarrow{i} \wedge \frac{d\overrightarrow{i}}{dt} \right\| \tag{S12}$$

$$v\, sin\alpha = D\left\| \begin{pmatrix} cos\delta \\ 0 \\ sin\delta \end{pmatrix} \wedge \begin{pmatrix} -\dot{\delta}\, sin\delta \\ \dot{\Phi}\, cos\delta \\ \dot{\delta}\, cos\delta \end{pmatrix} \right\| \tag{S13}$$

$$v\, sin\alpha = D\left\| \begin{pmatrix} -\dot{\Phi}\, sin\delta\, cos\delta \\ -\dot{\delta}\left(cos^2\delta + sin^2\delta\right) \\ \dot{\Phi}\, cos^2\delta \end{pmatrix} \right\| \tag{S14}$$

$$v\, sin\alpha = D\sqrt{\dot{\Phi}^2 sin^2\delta\, cos^2\delta + \dot{\delta}^2 + \dot{\Phi}^2 cos^4\delta} \tag{S15}$$

$$v\, sin\alpha = D\sqrt{\dot{\delta}^2 + \dot{\Phi}^2 cos^2\delta} \tag{S16}$$
