# Supplementary material for: Exploratory Movement Generates Higher-Order Information That Is Sufficient for Accurate Perception of Scaled Egocentric Distance
Source: PLoS One. 2015 Apr 9;10(4):e0120025. doi: 10.1371/journal.pone.0120025 (PMC4391914; doi:10.1371/journal.pone.0120025)
Supplement: S2 Text — Comparison of the accuracy of reachability judgments between participants who (according to Eq 13) most and least often generated accurate information about distance by moving orthogonal to the direction of the target. (PDF) [file pone.0120025.s006.pdf]

Exploratory movement generates higher-order information that is sufficient for accurate perception of scaled egocentric distance

Bruno Mantel, Thomas A. Stoffregen, Alain Campbell, Benoît G. Bardy

Supporting Information

Text S2

Could participants have taken advantage of the fact that the intermodal invariant specifying distance simplifies when their movement is orthogonal to the direction of the target (e.g., Eq. 10 simplifies into Eq. 13)? For each trial of the Movement condition, we computed the proportion of sampled points of head trajectory (i.e., time) during which each participant generated accurate information about distance (i.e., actual distance ±5cm) according to Eq. 13. We then split participants into two clusters by dividing this continuum dichotomously and tested whether those that generated more often accurate information through their movement were more accurate when judging whether the target was within reach.

Two participants were excluded from the analyses: the participant whose psychometric regression was not statistically significant, providing no consistent measure of $\mathrm{MR_P}$ (and thus of absolute error) and the participant at the median of the continuum, in order to have the same number of participants in each cluster ($N = 6$). The dichotomous division yielded a first cluster in which participants generated accurate distance information 37.4% of the time in average ($CI_{.95} = 30.4, 44.5\%$) and a second cluster for which this proportion was only 18.6% ($CI_{.95} = 14.7, 22.5\%$). As expected, the participants from the first cluster were more accurate when judging whether the target was within reach of their arm than those of the second cluster. In average, the absolute error of reachability judgments was 11.4% ($CI_{.95} = 6.5, 16.3\%$) for the participants of the first cluster whereas it was 30.9% ($CI_{.95} = 16.5, 45.2\%$) for the participants of the second. Mann-Whitney U tests indicated that this difference in judgments accuracy between the two clusters was statistically significant, $Z(N = 6) = 2.40, p < 0.05, d = 0.83$.
